# Cystinosis metabolic bone disease: inflammatory profile in human peripheral blood mononuclear cells and derived osteoclasts

**DOI:** 10.1007/s00431-024-05851-6

**Published:** 2024-11-14

**Authors:** Candide Alioli, Marcella Greco, Marie-Noëlle Méaux, Jérome Harambat, Rezan Topaloglu, François Nobili, Aurélia Bertholet-Thomas, Caroline Rousset-Rouviere, Aurélie Portefaix, Claire Dumortier, Francesco Emma, Irma Machuca-Gayet, Justine Bacchetta

**Affiliations:** 1 Pathophysiology, Diagnosis and Treatments of Bone Diseases, INSERM UMR1033, Lyon, France; 2 Department of Pediatric Subspecialties, Division of Nephrology, Bambino Gesù Children’s Hospital, IRCCS, Rome, Italy; 3 Reference Center for Rare Renal Diseases and Rare Diseases of Phosphate and Calcium, OSCAR and ORKID Networks, Hôpital Femme Mère Enfant, Bron, France; 4 Pediatric Nephrology Unit, Hôpital Pellegrin-Enfants, Clinical Investigation Center CIC-1401, Bordeaux, France; 5 Pediatric Nephrology Unit, School of Medicine, Hacettepe University, Ankara, Turkey; 6 Pediatric Department, CHU de Besançon, Besançon, France; 7 Pediatric Nephrology Unit, La Timone, University Hospital of Marseille, Marseille, France; 8 Clinical Investigation Center, Groupement Hospitalier Est, Hospices Civils de Lyon, Lyon, France; 9 Service de Néphrologie, Rhumatologie Et Dermatologie Pédiatriques, Hôpital Femme Mère Enfant, Boulevard Pinel, 69677 Bron Cedex, France

**Keywords:** Inflammation, Cystinosis, Bone, PBMCs, Osteoclasts

## Abstract

Cystinosis metabolic bone disease (CMBD) is an emerging concept in infantile nephropathic cystinosis, patients presenting with bone pains, fractures, and deformations during teenage or early adulthood. The underlying mechanisms remain unclear. Our aim was to explore the pro-inflammatory profile of osteoclastic lineage in cystinotic patients. We obtained blood samples from 14 cystinotic patients and 10 pediatric healthy controls. Peripheral blood mononuclear cells (PBMCs) were isolated and used to explore by RT-qPCR the transcript expression of 8 inflammatory markers (Il-6, Il-8, Il-1β, CXCL1, CCL2/MCP-1, CXCR3, Il-1 Receptor, Il-6 Receptor). In addition, when possible, PBMCs were differentiated into osteoclasts for further experiments. The expression of Il-6, IL-8, CXCR3, and CCL2/MCP-1 was significantly increased in PBMCs from cystinotic patients. We also explored the expression of Il-1 Receptor and Il-6 Receptor, two major pro-osteoclastic signal inducers, in osteoclasts differentiated from PBMCs from controls (*N* = 3) and patients (*N* = 4). The expression of IL-1 Receptor (but not IL-6 receptor) was increased in osteoclasts obtained from cystinotic patients.

*Conclusion*: There is an inflammatory profile in PBMCs and osteoclastic lineage in cells obtained from cystinotic patients. CXCR3 and MCP-1 stimulate migration and activation of macrophages, that may explain the previously reported local increased osteoclastogenesis. The osteoclastic overexpression of IL-1 Receptor is a relevant observation in the field since blocking Il-1β signaling has recently been proposed as a novel therapeutic approach to improve muscular wasting in this orphan disease.

**Table:** 

**What is Known:**	
• *Cystinosis metabolic bone disease (CMBD), an emerging concept with unclear underlying mechanisms, induces bone pains, fractures and deformations in patients with cystinosis.*	
• *Blocking Il-1β signaling may be a novel therapeutic approach to improve muscular wasting in cystinosis.*	
**What is New:**	
• *There is an inflammatory profile in PBMCs and osteoclastic lineage in cells obtained from cystinotic patients, with an over-expression of IL-1 Receptor in osteoclasts.*	
• *We provide another experimental rationale to propose targeted anti-inflammatory therapies in cystinotic patients with severe bone disease.*

**Supplementary Information:**

The online version contains supplementary material available at 10.1007/s00431-024-05851-6.

## Introduction

Nephropathic cystinosis (NC) is a rare autosomal recessive disease secondary to impaired activity of cystinosin, a lysosomal H + /cystine symporter encoded by the *CTNS* gene. Systemic accumulation of cystine crystals leads to progressive tissue damage, starting from the kidney. Patients develop early-onset proximal tubulopathy and progress to end-stage kidney failure (ESKF) by 10 years of life if untreated. Cysteamine therapy was introduced in the 1990s and allows postponing ESKF by approximately 10 years, as well as other extrarenal comorbidities [1].

Cystinosis metabolic bone disease (CMBD) indicates a dysregulation of bone metabolism observed specifically in NC. Following initial reports describing intrinsic cystinotic bone cell defects [2–5], CMBD has been formally recognized as a specific complication of NC (on top of typical mineral and bone disorders associated with chronic kidney disease) in an international consensus document published in 2019, with recommendations for both diagnosis and management [6]. CMBD has a significant impact on quality of life, with an increased frequency of bone pain, deformities, and fractures, especially during late adolescence and early adulthood. Biochemically, patients present with low circulating levels of fibroblast growth factor 23 (FGF23) and parathyroid hormone (PTH) [7], likely resulting (at least partly) from tubular phosphate leak and in contrast to patients with chronic kidney disease (CKD) with a different primary diagnosis.

We previously showed increased osteoclastic differentiation and resorption using human bone biopsies [5]. In addition, we observed that peripheral blood mononuclear cells (PBMCs) obtained from patients with cystinosis were more prone to generate osteoclasts compared to cells obtained from healthy donors [3]. However, the underlying mechanisms to explain these findings remain unclear. Given that several inflammatory pathways have been identified in non-osseus experimental models of NC [8, 9], and recognizing that osteoclastogenesis is enhanced by inflammatory cytokines, we have explored the pro-inflammatory profile of osteoclastic lineages obtained from patients with NC.

## Methods

### Clinical study

The CYSTEABONE study (NCT03919981) is a prospective multicenter clinical study aimed at analyzing in vitro the osteoclastic activity of PMBC-derived cells obtained from patients with genetically confirmed NC aged over 2 years. In addition to routine laboratory tests, and after informed consent, we obtained total blood samples to analyze osteoclastic differentiation. The CYSTEABONE study was approved by the *Comité de Protection des Personnes Sud-Méditerranée* IV (2019-A00166-51) and by local IRBs of collaborating centers, in accordance with the Declaration of Helsinki. Informed consent was obtained from all individual participants included in the study.

We had previously observed positive genotype/phenotype correlation at the osteoclastic level using samples from 17 cystinotic patients [10]. In this study, we obtained blood samples from 14 additional cystinotic patients and 10 healthy controls taken from [11]; pediatric controls were out-clinic patients. The exclusion criteria to be a control were CKD or other pro-inflammatory diseases or recent infection, diseases with known abnormal FGF23 levels, oral corticosteroids during the last 3 months, and undergoing immunosuppressive therapies.

In patients, the estimated glomerular filtration rate (eGFR) was calculated using the 2009 revised Schwartz formula, and phosphate, height, and body weight were standardized on age (and sex for anthropometric variables, on French growth charts). Expression by PBMCs of 8 inflammatory markers before and after osteoclast differentiation was analyzed by RT-qPCR. Blood samples were drawn fasting before the administration of cysteamine.

### Peripheral blood mononuclear cells obtention

Blood sample volumes ranged from 15 to 30 mL, in accordance with the French legislation on the maximal blood sample volume that can be collected for human research [11]. Once patients accepted to participate, we organized the shipping of fresh samples at room temperature within 24 to a maximum of 48 h to the lab using a professional transporter, since we had previously shown that we can perform osteoclastogenesis up to 48 h after sampling (unpublished personal data). Upon arrival, PBMCs were immediately isolated by density gradient centrifugation using Lymphocyte Separating Medium (Ficoll, Eurobio ®), as previously published [11].

### Reverse transcription and real-time quantitative PCR (RT-qPCR)

RNA was extracted from PBMCs using trizol reagent (Invitrogen) followed by purification with the Macherey–Nagel® purification kit, according to the manufacturer’s instructions. After reverse transcription (iScript cDNA synthesis kit Biorad), quantitative PCR was performed using Itaq® enzyme and primers specific for each exon sequence. All tests were performed in duplicates. Results were normalized for hGAPDH expression and expressed as relative values using the 2^(-ΔCq) method. The expression of the following genes, all involved in inflammation and chemotaxis, was measured: IL1β, IL6, IL8, CXCL1, CCL2 (also named MCP1), IL1R, IL6R, and CXCR3.

### Osteoclastic differentiation

If enough cells were obtained, PBMCs were seeded on 96-well plates or on 6-well plates at 37 °C in α-Minimum Essential Medium (α-MEM, Life technologies®) supplemented with L-Glutamine (2 mM, Gibco®) and penicillin/streptomycin (50 µg/mL streptomycin, Gibco®), enriched with 10% FBS (Sigma-Aldrich®) as previously described in the literature [11]. They were differentiated into osteoclasts using M-CSF at 20 ng/mL (PeproTech®) and RANK-L at 40 ng/mL (PeproTech ®), medium and cytokines being changed every 2 or 3 days. At the end of the differentiation, cells were fixed with 4% Paraformaldehyde (PFA). Osteoclast differentiation was evaluated by cell staining with a tartrate-resistant acid phosphatase (TRAP) kit (ref 387A, Sigma-Aldrich®). The expression of inflammation genes after osteoclast differentiation was evaluated by RT-qPCR, as described above.

### Statistical analyses

Statistical analyses were performed using GraphPad Prism 8 software. The non-parametric Mann–Whitney test was used for comparisons. Results are expressed as median (IQR). All tests are two-sided and considered significant for a *p* value < 0.05.

## Results

Blood samples were obtained from 14 cystinotic patients and 10 healthy controls (Supplemental Table). The patients were evaluated at a median age of 8.4 (8.3) years, and diagnosis was established at 1.4 (0.6) years; they were all on conservative therapy, and none had a past of neither dialysis nor transplantation. Standardized height and body weight for age and sex were − 1.5 (1.4) and − 0.4 (1.2), respectively. The daily dose of cysteamine was 38 (12) mg/kg; of note, three patients were receiving delayed-release cysteamine, and the other 11 cysteamine. The average hemicystin levels (i.e., white blood cells cystine level used for routine follow-up of cystinotic patients, target < 1) during the year preceding inclusion was 1.1 (0.8) µmol/g proteins. Biochemical results at the time of sample are displayed in the Supplemental Table. Renal function was rather preserved since 10 out of 14 patients displayed eGFR above 45 mL/min/1.73 m^2^.

**Fig. 1:**
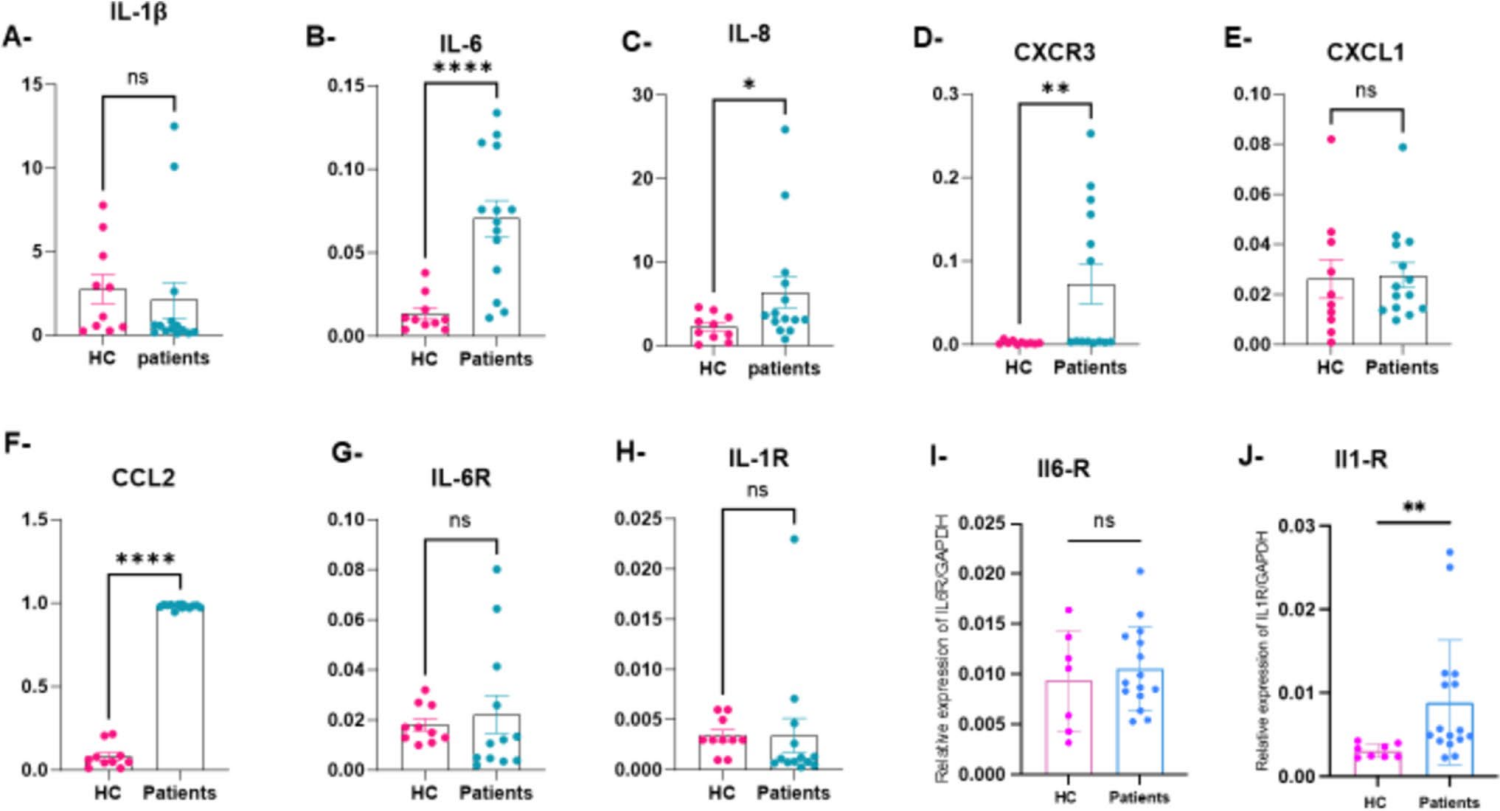
Expression of the different inflammatory cytokines in PBMCs and osteoclasts. HC, healthy controls; results of RT-qPCR for the 8 genes involved in inflammation and chemotaxis (IL1β, IL6, IL8, CXCL1, CCL2 (also named MCP1), IL1R, IL6R, CXCR3) in PBMCs from 14 patients and 10 controls (**A**–**H**), and in fully-differentiated osteoclasts from 4 patients and 3 controls (I–**J**)*.* Gene expression was quantified and normalized to hGAPDH values, then expressed as relative expression using the 2^(-ΔCq) method. *p < 0.01 and **p < 0.001

No significant differences were observed in the PMBC expression of IL1 Receptor, IL-6 receptor, Il1β, and CXCL1. Conversely, the expression of Il-6 (a key pro-inflammatory cytokine), IL-8, CXCR3, and CCL2/MCP-1 was significantly increased in PMBCs obtained from cystinotic patients (Fig. 1A–H**)**.

To investigate further whether osteoclasts derived from cystinotic patients responded more to pro-inflammatory signals, we analyzed the expression of Il-1 Receptor and Il-6 Receptor, two major pro-osteoclastic signal inducers independently from the canonical RANKL/OPG pathway. After PMBC differentiation into osteoclasts, the expression of IL-1 Receptor but not of IL-6 Receptor was increased in cells obtained from patients (*N* = 4), compared to cells obtained from controls (*N* = 3) (Fig. 1I–J**)**.

## Discussion

Our results show a distinct inflammatory profile in PBMCs and osteoclastic lineages obtained from cystinotic patients when compared to healthy controls. Inflammation may be seen in patients with CKD from other causes, especially at the dialysis and post-transplantation stage, but, in our study, all patients were under conservative therapy with rather preserved and/or near-normal renal function. The fact that the healthy controls were initially used for a study on another rare disease namely X-linked hypophosphatemia (and matched on age and gender in that setting) may induce a bias [11], especially since we have few details. However, it is challenging to obtain samples from healthy controls and we analyzed the same 8 genes at a similar period of time, with the same assays for RT-qPCR. As expected, patients with cystinosis had significant growth retardation and decreased renal function (however, with rather preserved renal function for this disease) as compared to controls, but age was not different, as well as ALP, PTH, and 25 OH vitamin D levels.

CXCR3 and MCP-1 stimulate migration and activation of macrophages [12]. IL-6 signaling in bone cell physiology affects osteoclasts, but also osteoblasts and osteocytes by complex mechanisms involving also IL-6 soluble receptor, that are not completely understood. Here, we did not address this question, but we did not observe significant differences on IL-6 receptor spliced variants targeted transcripts. However, in humans, high levels of IL-6 are associated with bone loss after menopause.

Overall this may explain the locally increased osteoclastogenesis that was previously observed in human bone biopsies in cystinosis [5]. CXCR3, also termed CD183, is preferentially expressed in TH1 cells. It plays a role in integrin activation and chemotactic migration, whereas MCP-1 recruits monocytes, memory T cells, and dendritic cells into inflammatory tissues. In bone, MCP-1 is expressed in mature osteoblasts and osteoclasts, under the control of nuclear factor κB. Both CXCR3 and MCP-1 are known to be increased in chronic inflammatory diseases (e.g., rheumatoid arthritis). Increase in CXCR3 and MCP-1 in cystinosis has not been previously reported to our knowledge.

We did not observe differences in PMBC Il1 expression, but osteoclastic overexpression of IL-1 Receptor is in line with previous reports showing increased circulating Il1 (and subsequent Il18) levels in cystinotic patients, compared to patients with Familial Mediterranean fever, probably due to endogenous inflammasome activation by cystine crystals [8]. Accordingly, Il-1β signaling blockade has also been proposed as a potential therapy to improve muscle wasting in a murine model of cystinosis [9].

Taken together, these results partly explain the complexity of CMBD and suggest that targeted anti-inflammatory therapies may be clinically helpful in cystinotic patients with severe bone disease.

## Supplementary Information

Below is the link to the electronic supplementary material.

Supplementary file1 (DOCX 27 KB)

## Data Availability

The datasets generated during and/or analyzed during the current study are not publicly available due to confidential data but are available from the corresponding author on reasonable request.

## Abbreviations

CMBD: Cystinosis metabolic bone disease
CKD: Chronic kidney disease
eGFR: Estimated glomerular filtration rate
FGF23: Fibroblast growth factor 23
NC: Nephropathic cystinosis
PBMC: Peripheral blood mononuclear cells
PTH: Parathyroid hormone
SDS: Standard deviation score
