# Supplementary material for: Cystinosis metabolic bone disease: inflammatory profile in human peripheral blood mononuclear cells and derived osteoclasts
Source: Eur J Pediatr. 2024 Nov 14;184(1):9. doi: 10.1007/s00431-024-05851-6 (PMC11564333; doi:10.1007/s00431-024-05851-6)
Supplement: Supplementary file 1 — Supplementary file1 (DOCX 27 KB) [file 431_2024_5851_MOESM1_ESM.docx]

**Supplemental Table: comparison between controls and patients with cystinosis**

|  | **Patients with cystinosis**  **N=14** | **Controls**  **N=10** |
| --- | --- | --- |
| **Age (years)** | 8.4(3.3-16.0) | 11.5(4.0-17.0) |
| **Height (SDS)*** | -1.5(-3.1 ;-0.2) | 0.8(-1.0 ;2.0) |
| **Body weight (SDS)*** | -0.4(-2.5 ;0.2) | 1.3(-1.5 ;3.5) |
| **eGFR (ml/min/1.73 m²)*** | 61(20-110) | 115(96-209) |
| **Ca (mmol/L)*** | 2.29(2.19-2.54) | 2.45(2.33-2.68) |
| **Phosphate (mmol/L)** | 1.34(0.77-1.53) | 1.36(1.06-1.54) |
| **ALP (UI/L)** | 394(77-808) | 228(104-320) |
| **PTH (ng/L)** | 43(7-168) | 33(21-82) |
| **25OH vitamin D (nmol/L)** | 92(47-126) | 67(51-111) |
| **Time of sampling** | March to November 2021 | May to September 2021 |

Non-parametric Mann Whitney tests were performed, *: p<0.05. Results are expressed as median(range). Pediatric controls were seen at the outpatient clinics for the following reason: follow-up of urolithiasis (N=2), follow-up of congenital abnormality of the kidney and urinary tract (N=4), chronic follow-up of nephrotic syndrome without treatment/relapse of hemolytic and uremic syndrome after the acute phase (N=3), miscellaneous (N=1). Of note, all controls had C Reactive Protein below 1 mg/L. eGFR: estimated glomerular filtration rate calculated with the 2009 Schwartz formula.
